# Development and validation of an individualized gene expression-based signature to predict overall survival of patients with high-grade serous ovarian carcinoma

**DOI:** 10.1186/s40001-023-01376-0

**Published:** 2023-10-27

**Authors:** Dandan Yuan, Hong Zhu, Ting Wang, Yang Zhang, Xin Zheng, Yanjun Qu

**Affiliations:** 1 Department of Obstertrics and Gynecology, The Second Affiliated Hospital of Harbin Medical University, Harbin, 150086 China; 2 Department of Gynecological Oncology, Renji Hospital Affiliated to Medical College of Shanghai Jiaotong University, Shanghai, 200000 China; 3 Department of Hepatological Surgery, The Third Affiliated Hospital of Harbin Medical University, Harbin, 150001 China; 4 Department of Gynecology, The First Hospital of Jiaxing City, Jiaxing, 314000 China; 5 Department of Obstertrics and Gynecology, The First Affiliated Hospital of Harbin Medical University, Harbin, 150001 China

**Keywords:** The Cancer Genome Atlas, Bioinformatics, Gene Expression Omnibus, Prognostic markers

## Abstract

**Background:**

High-grade serious ovarian carcinoma (HGSOC) is a subtype of ovarian cancer with a different prognosis attributable to genetic heterogeneity. The prognosis of patients with advanced HGSOC requires prediction by genetic markers. This study systematically analyzed gene expression profile data to establish a genetic marker for predicting HGSOC prognosis.

**Methods:**

The RNA-seq data set and information on clinical follow-up of HGSOC were retrieved from Gene Expression Omnibus (GEO) database, and the data were standardized by DESeq2 as a training set. On the other hand, HGSOC RNA sequence data and information on clinical follow-up were retrieved from The Cancer Genome Atlas (TCGA) as a test set. Additionally, ovarian cancer microarray data set was obtained from GEO as the external validation set. Prognostic genes were screened from the training set, and characteristic selection was performed using the least absolute shrinkage and selection operator (LASSO) with 80% re-sampling for 5000 times. Genes with a frequency of more than 2000 were selected as robust biomarkers. Finally, a gene-related prognostic model was validated in both the test and GEO validation sets.

**Results:**

A total of 148 genes were found to be significantly correlated with HGSOC prognosis. The expression profile of these genes could stratify HGSOC prognosis and they were enriched to multiple tumor-related regulatory pathways such as tyrosine metabolism and AMPK signaling pathway. AKR1B10 and ANGPT4 were obtained after 5000-time re-sampling by LASSO regression. AKR1B10 was associated with the metastasis and progression of several tumors. In this study, Cox regression analysis was performed to create a 2-gene signature as an independent prognostic factor for HGSOC, which has the ability to stratify risk samples in all three data sets (*p* < 0.05). The Gene Set Enrichment Analysis (GSEA) discovered abnormally active REGULATION_OF_AUTOPHAGY and OLFACTORY_TRANSDUCTION pathways in the high-risk group samples.

**Conclusion:**

This study resulted in the creation of a 2-gene molecular prognostic classifier that distinguished clinical features and was a promising novel prognostic tool for assessing the prognosis of HGSOC. RiskScore was a novel prognostic model which might be effective in guiding accurate prognosis of HGSOC.

**Supplementary Information:**

The online version contains supplementary material available at 10.1186/s40001-023-01376-0.

## Introduction

In the Western world, epithelial ovarian cancer (EOC) is one of the major contributors to gynecological mortalities [1]. EOC, a heterogeneous tissue consisting of several tumor subtypes, shows different genetic risks, pathophysiology, clinical behaviors, responses to treatment, and prognosis. High-grade serous ovarian cancer (HGSOC) constitutes 60% -70% of all EOC [2], and the majority of the EOC deaths are caused by HGSOC [3]. Currently, BRCA1/BRCA2 gene mutation, family history, non-fertility, use of oral contraceptives, fallopian tube ligation, pregnancy, and lactation are seen as risk factors for ovarian cancer [4]. Tumor resection, platinum, and taxane chemotherapy are common options for treating ovarian cancer [5]. Since a significant number of HGSOC patients are identified at advanced stages, they have a higher recurrence rate and the 5-year rate of survival for these patients is < 40% [6, 7]. Identifying non-responders and patients with primary platinum resistance plays a crucial role in achieving a better survival of HGSOC patients [7]. As a result, it is critical to identify prognostic biomarkers to provide a reference for personalized medicine and improve the prediction of clinical outcomes.

With advances in sequencing technology, it has been possible to explore the molecular mechanisms of disease by mapping the genomes of cancer cases [8]. Many of the biomarkers and mechanisms have contributed to a deeper understanding of cancer [9, 10]. Numerous studies have been conducted to develop biomarkers for survival prediction and the long-term prognosis of HGSOC. By analyzing high-throughput gene expression profiles, genetic markers constructed with several to dozens of prognostic genes could effectively predict total survival [11, 12], reduce the status of the product [13] and platinum treatments [14]. For HGSOC patients with extreme chemical reactions, Wisman GBA et al. [15] applied genome-wide analysis of DNA methylation to construct new HGSOC platin-sensitive epigenetic markers. According to the transcriptome data, Liu L et al. [16] screened seven genes of new signal prediction based on high IIIc serous ovarian cancer clinical outcome and cisplatin sensitivity. However, there are currently no effective clinical biomarkers for predicting HGSOC patients’ response to treatment. Even with relevant research, there are too many biomarkers identified, and there is a certain operational complexity in clinical application. Thus, identifying genetic signals related to the prognosis of HGSOC by analyzing its biological functions through bioinformatics should be studied.

In the present research, to effectively construct a reliable gene signature for predicting the prognosis of patients with HGSOC, a systematic pipeline was proposed to screen HGSOC-related genetic markers, and gene expression profiles of HGSOC patients were obtained from Gene Expression Omnibus (GEO) and The Cancer Genome Atlas (TCGA) databases. Screening of prognostic markers was performed combining transcriptome and genomics data, eventually constructing a 2-gene signature. It was found that performance in predicting survival rate was validated by external validation sets and test sets. The current findings revealed that the 2-gene signatures were involved in important pathways and biological processes of HGSOC, indicating that the 2-gene signature can be utilized in the prediction of prognostic risk among HGSOC patients, and provision of baseline information for molecular mechanism comprehension of the prognosis of patients with HGSOC. We provided a prospective scientific basis for prognostic guidance and in-deep exploration of the pathogenesis of HGSOC.

## Materials and methods

### Data acquisition and processing

**Fig. 1:**
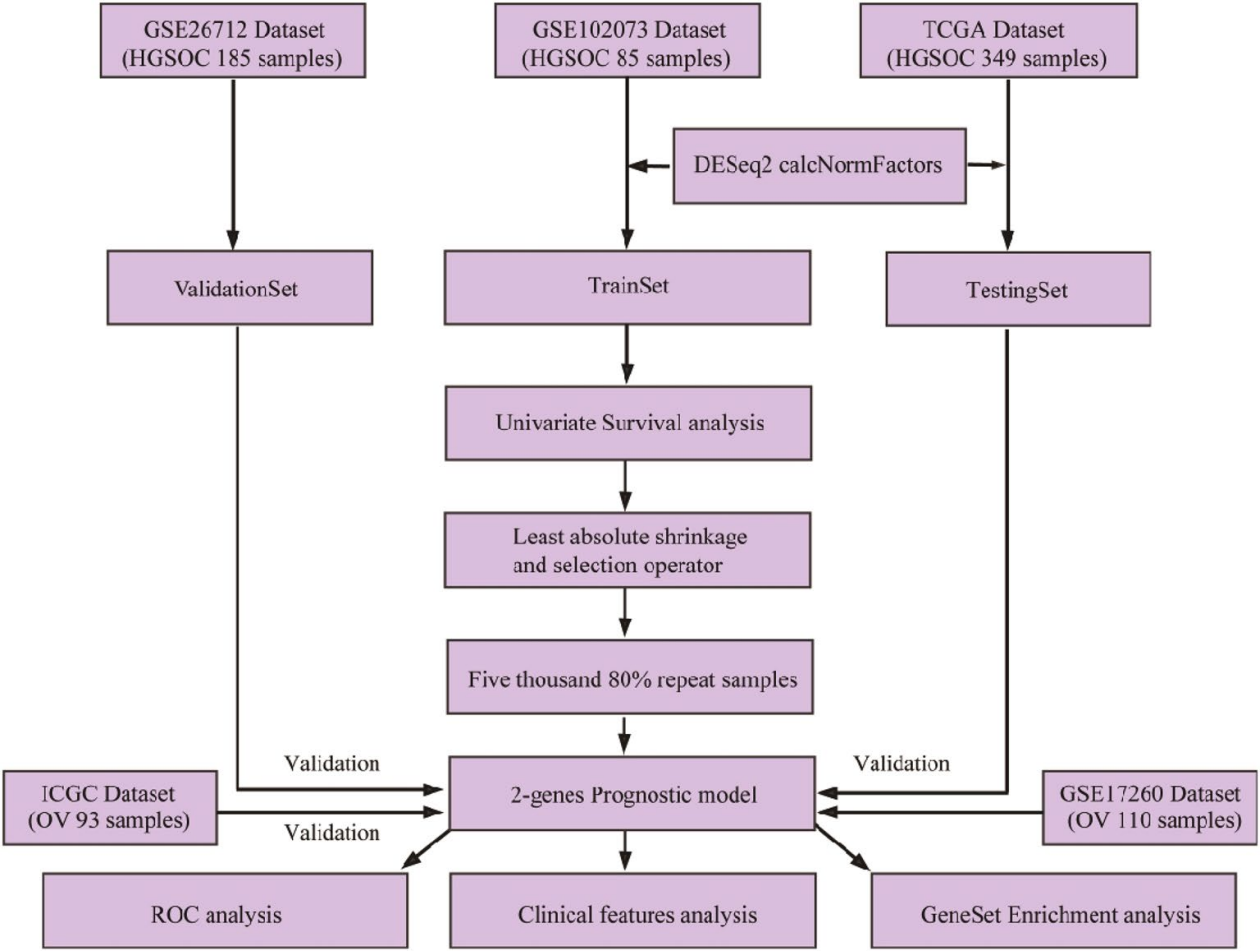
Work flowchart

The gene expression profile of HGSOC GSE102073 contained primary tumor tissue samples from eight-five patients diagnosed with HGSOC. These samples were downloaded from the GEO database on Illumina HiSeq 2500 platform [17]. The samples were used as training sets and clinical information of the data set was from Ducie J [17]. In April 2019, RNA-seq data (counts) contained 371 ovarian cancer samples that were obtained from the TCGA database (https://cancergenome.nih.gov/) as a test set. Three hundred and forty-nine samples of HGSOC patients with a follow-up period of longer than 30 days were extracted (see Table 1). Unified data standardization in the validation set and the training set was conducted using the CalcNormFactors function of R software package DESeq2 [18] to filter genes with low expression abundance. The genes with a count sum < 20 in all training set samples were eliminated, and 18,738 genes with high expression abundance were obtained. In addition, to verify the cross-platform nature of data, GSE26712 [19] of the Affymetrix Human Genome U133A Array platform was used as an external validation set. The specific information is shown in Table 1. Figure 1 shows the flowchart.

**Table 1:** Clinical information statistics of training set and validation set samples

Characteristic	GSE102073 training datasets (*n* = 85)	TCGA test datasets (*n* = 371)	GSE26712 validation set (*n* = 185)
Age			
≤ 50	62	283	NA
> 50	11	75	NA
Stage			
Stage III	49	285	NA
Stage IV	20	53	NA
Other	4	20	NA
Survival status			
Live	17	160	24
Dead	56	189	129

### Univariate Cox proportional hazard regression analysis

Following Jin-Cheng et al. [20], we utilized the R package survival coxph function [21] to conduct univariate Cox proportional hazards regression analysis on every gene for the purpose of screening those that were remarkably related to the patient's OS in the training data set (*p* < 0.01 was the threshold). Furthermore, unsupervised cluster analysis was conducted based on the expression profiles of prognostic-related genes to determine the classification and prognostic differences of the samples.

### Construction of a prognostic immune gene signature

The selected genes were significantly related to the patient’s OS. The least absolute shrinkage and selection operator (LASSO) [22] regression algorithm was utilized for dimension reduction analysis. LASSO approach is also used with the Cox model for analysis of survival. At present, it has been effectively used to generate sparse signatures for survival prediction in a variety of fields such as oncology [23–25]. The R software package Glmnet [26] was utilized to screen prognostic characteristic genes, and the optimal characteristics were analyzed by tenfold cross-validation. To obtain robust results, 80% of the samples were subjected to regression analysis, and 5,000 repeated samples were put back to analyze and calculate the frequency of gene selection. As genes with a high frequency were more likely to be stable prognostic genes, those genes with occurrence frequency greater than 2000 times were determined as stable characteristic genes. The relationship between the expression of each potential characteristic gene and prognosis was examined by ROC analysis. Multivariate Cox regression analysis was conducted for this study. The constructed risk scoring model is illustrated below:

$${\text{RiskScore}} = \mathop \sum \limits_{k = 1}^{N} {\text{Exp}}_{k} *e^{{{\text{HRK}}}} ,$$

where *N* denotes the number of prognostic genes, ${\mathrm{Exp}}_{k}$ denotes the expression value of the prognostic genes, and $e^{{{\text{HRk}}}}$ denotes the estimated value of the regression coefficient of the genes in a multivariate Cox regression analysis.

### Functional enrichment analyses

Kyoto Encyclopedia of Genes and Genomes (KEGG) and Gene Ontology (GO) analysis of pathway enrichment was conducted using the R package clusterprofiler [27] for the purpose of determining the over-represented Gene Ontology terms in 3 groups (cellular component, molecular function, and biological processes) and KEGG pathway. In the present research, FDR < 0.05 was considered to be statistically significant.

The MSigDB26 was used to perform gene set enrichment analysis (GSEA)^25^ on the C2 canonical pathways gene set collection (with 1320 gene sets) with the aid of the JAVA software (http://software.broadinstitute.org/gsea/downloads.jsp) [28]. After conducting 1000 permutations, gene sets with a false discovery rate (FDR) value no greater than 0.05 were considered to be significantly enriched.

### Statistical analysis

Kaplan–Meier (KM) curves were plotted for the purpose of assessing the risk of survival between the low and high-risk groups of patients with HGSOC. In addition, the median risk score of each individual data set was used as the cut-off. The independence of gene markers as prognostic variables was investigated by means of multivariate Cox regression analysis. Significance was defined by *p* values < 0.05. Moreover, R (version: 3.4.3) was used to conduct all of the analyses.

## Results

### Identification of HGSOC survival-associated gene sets

**Fig. 2:**
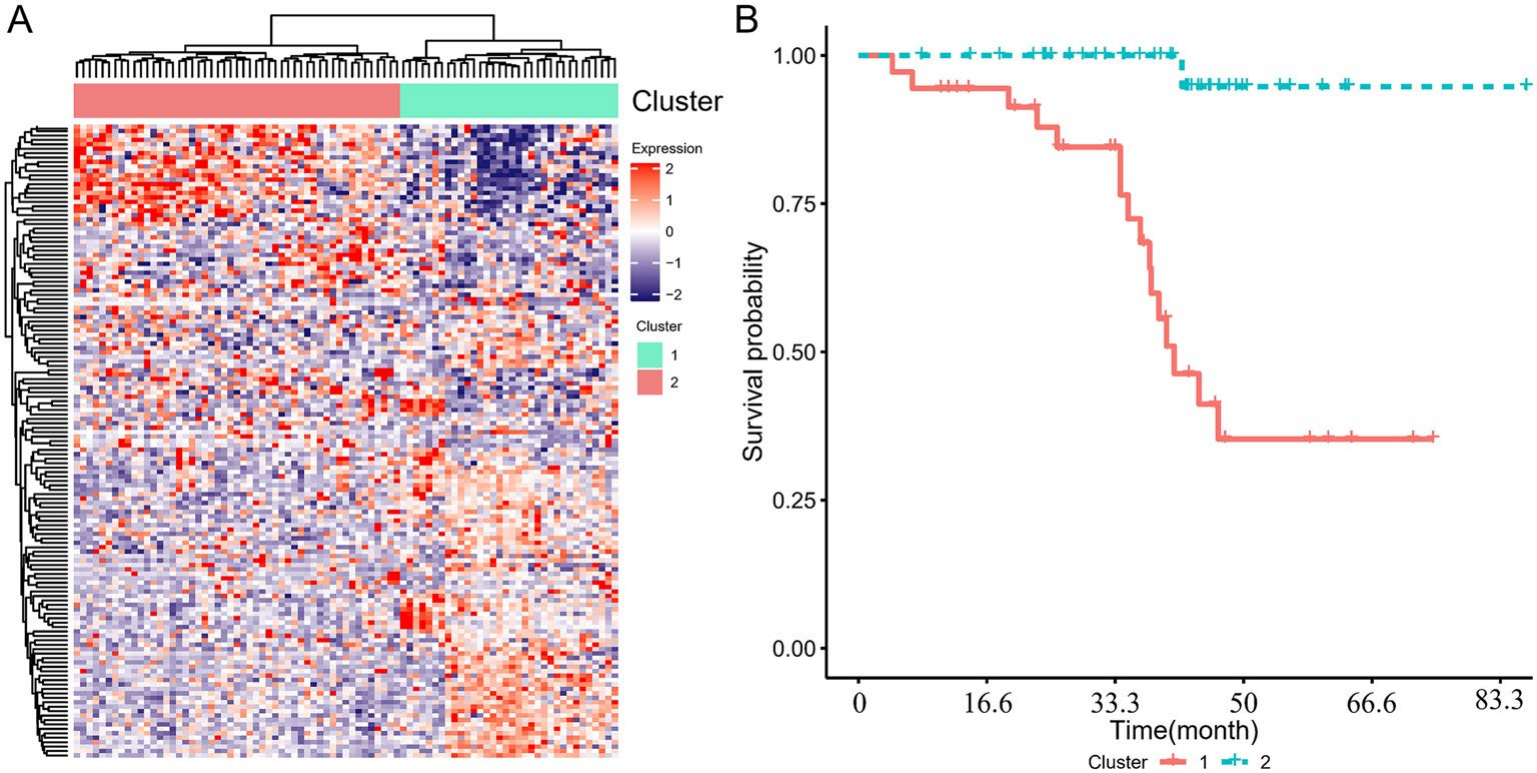
Identification of molecular subtypes. **A** Clustering heat map of expression profiles of prognosis-related genes, with the horizontal axis representing samples and the vertical axis representing genes, the reddish color indicates higher expression. **B** Kaplan–Meier (KM) curve of the prognostic difference between Cluster1 and Cluster2

In the samples of the GSE102073 training set, univariate regression analysis was used to analyze the relationship between gene expression and overall survival (OS). 148 univariate genes incorporating 120 genes with HR > 1 and 28 genes with HR < 1 were identified through Cox regression log-rank with *p*-value ≤ 0.01 (Table 2). Based on the close relationship between these genes and prognosis, the expression profile of these genes was utilized in hierarchical clustering analysis on ovarian cancer patients, and it was observed that these genes could classify the patients into two groups (Cluster1: *N* = 31, Cluster2: *N* = 42) (Fig. 2A). 12 cases (38.7%) died in Cluster1, and only 1 case (11.9%) died in Cluster2, demonstrating that there is a remarkable difference between the 2 groups (*p*= 0.016475). The prognosis of patients in the Cluster1 group was remarkably poorer compared to patients in the Cluster2 group, according to further examination of the prognostic differences between the two groups of samples (*p*< 0.001) (Fig. 2B), indicating that the prognosis of patients with HGSOC may be accurately stratified using the 148 gene expression profile.

**Table 2:** The top20 genes most relevant to OS

Genes	HR	(95%_CI_for_HR)	*p* value
APOC2	240	(16–3600)	7.60E−05
GOLGA8Q	58	(6.1–550)	0.00041
CRYAB	1.8	(1.3–2.4)	0.00048
LSAMP	3.3	(1.7–6.4)	0.00049
CDRT15	59	(5.7–610)	0.00063
SLC6A3	2.9	(1.6–5.4)	0.00064
PMFBP1	9.3	(2.6–33)	0.00066
ASIC2	2.8	(1.6–5.2)	7.00E−04
KRT80	1.7	(1.2–2.3)	0.00082
GP6	13	(2.8–63)	0.001
GALP	9.8	(2.5–38)	0.001
LY6D	2.9	(1.5–5.4)	0.0012
CATSPERG	3.3	(1.6–7)	0.0013
RASGRP4	10	(2.5–42)	0.0014
FAM127B	0.25	(0.11–0.58)	0.0014
DLX2	9	(2.3–35)	0.0015
LGALS14	5.9	(2–18)	0.0015
BTNL8	2.7	(1.4–4.9)	0.0016
OR2AG1	21	(3.1–140)	0.0017
CSTB	0.33	(0.17–0.66)	0.0017

### Identification of robust prognostic factors

**Fig. 3:**
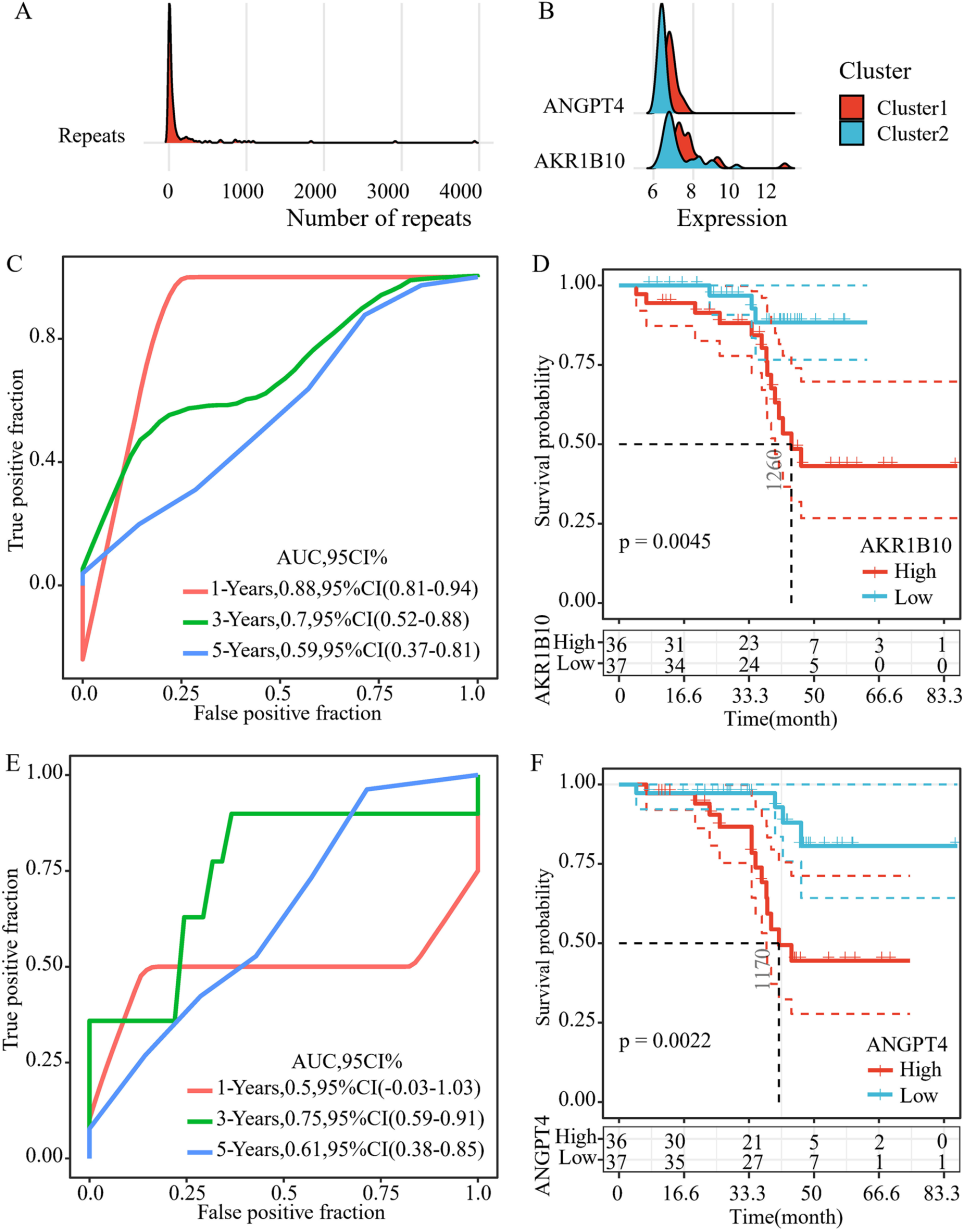
Identification of robust prognostic factors. **A** The frequency of genes in five thousand lasso regressions. **B** The difference in the expression distribution of the two genes in Cluster1 and Cluster2. **C** 1, 3and 5 years AUC of ROC of AKR1B10. **D** KM survival curve of high expression of AKR1B10 group and low expression of AKR1B10 group. **E** 1-, 3-, and 5- years AUC of ROC of ANGPT4. F: KM survival curve of high expression of ANGPT4 group and low expression of ANGPT4 group

Here, 148 genes related to the prognosis of HGSOC have been identified. We further limited the range of these genes while ensuring high accuracy. LASSO Cox regression analysis was performed with the aid of the R software package Glmnet, and the frequency distribution of gene occurrence yielded two genes with occurrence frequency higher than 2000 (AKR1B10 and ANGPT4) (Fig. 3A). The two genes showed significantly different expressions in Cluster1 and Cluster2, with a mean value higher in Cluster1 than in Cluster2 (Fig. 3B). Three-year AUC values of the two genes reached 0.7 (Fig. 3C, E). The median was set as the cut-off value with the aim of analyzing the prognostic differences between patients with high expression and low expression of AKR1B10 and ANGPT4. The results demonstrated that the prognosis of patients with high-expressed AKR1B10 and ANGPT4 was significantly more unfavorable than those in the low-expression group (*p*< 0.01) (Fig. 3D, F).

### Identification of a 2-gene signature for HGSOC survival

By performing multivariate Cox regression analysis, a 2-gene signature associated with the prognosis of patients with HGSOC based on AKR1B10 and ANGPT4 was constructed. The model was as follows:

$${Risk}_{2}=0.567*AKR1B10+1.331*ANGPT4.$$

**Fig. 4:**
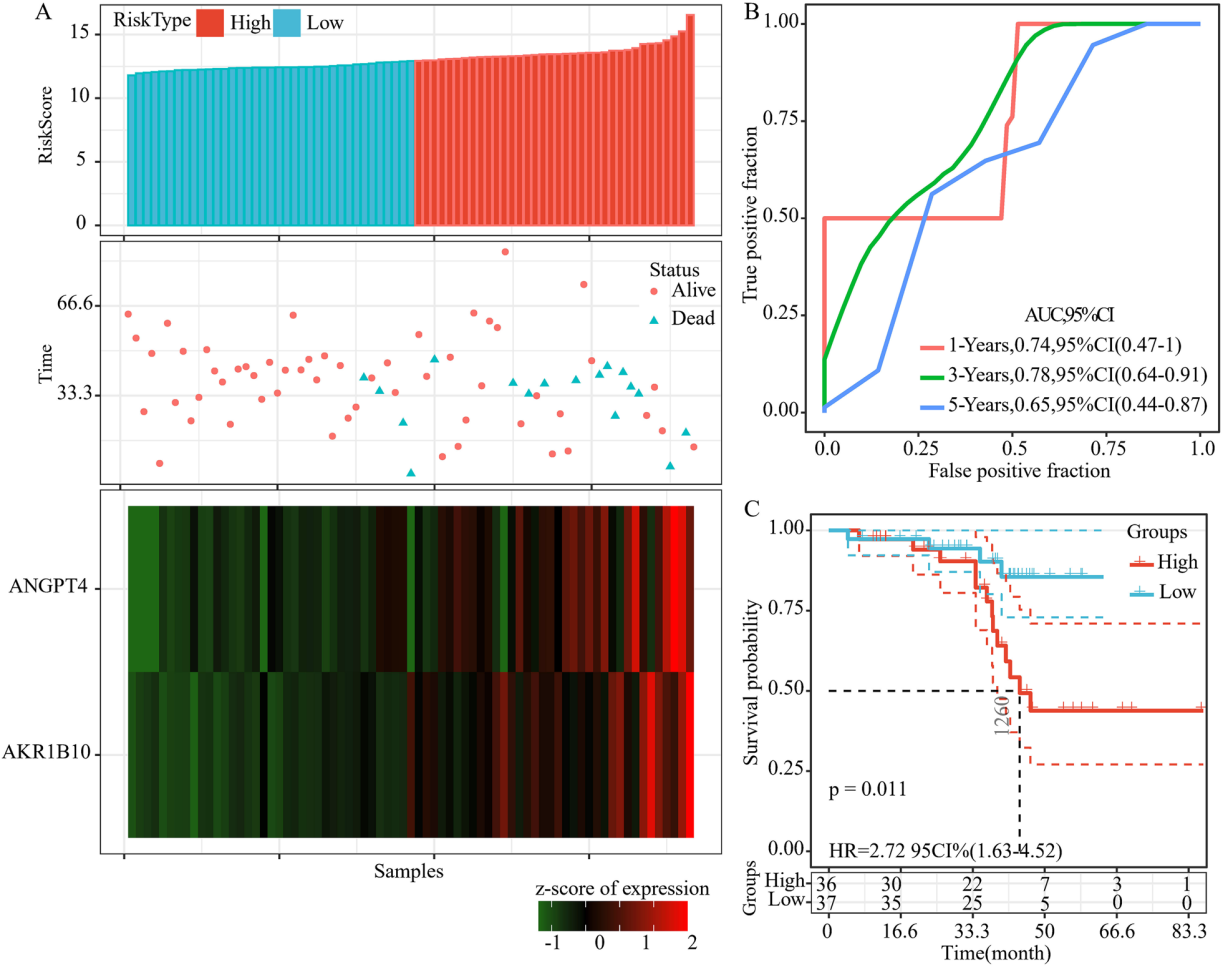
The relationship between risk score and prognosis. **A** The relationship between 2-gene signature and survival status, survival duration, and expression in the training set samples. **B** ROC and AUC of 2-gene signature. **C** KM survival curve of 2-gene signature in the training set

The risk score in each sample was determined in the training set. We discovered that a greater risk score was associated with a shorter survival duration, and that the expression levels of AKR1B10 and ANGPT4 were elevated (Fig. 4A). Furthermore, the average 1, 3, and 5-year AUC values of the 2-gene signature reached 0.7 (Fig. 4B). Finally, the samples were classified into two groups based on the median risk score, and we discovered that patients in the low-risk group and the high-risk group had dramatically different prognoses (Fig. 4C).

### Robustness of the 2-gene signature model

**Fig. 5:**
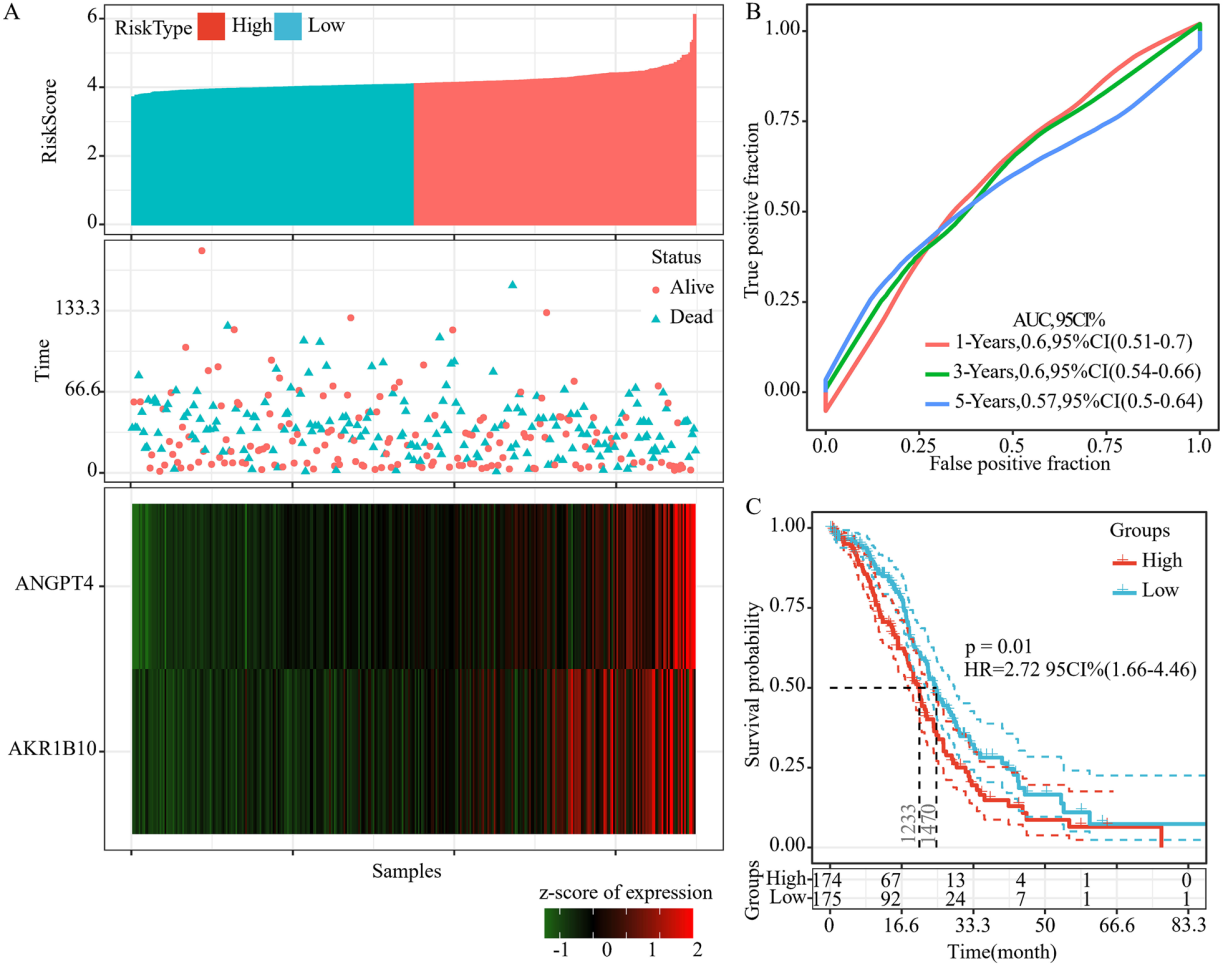
The relationship between risk score and prognosis in TCGA test set. **A** The relationship between 2-gene signature and survival status, survival duration, and expression in TCGA test set. **B** ROC and AUC of 2-gene signature in TCGA test set. **C** KM survival curve of 2-gene signature in TCGA test set

**Fig. 6:**
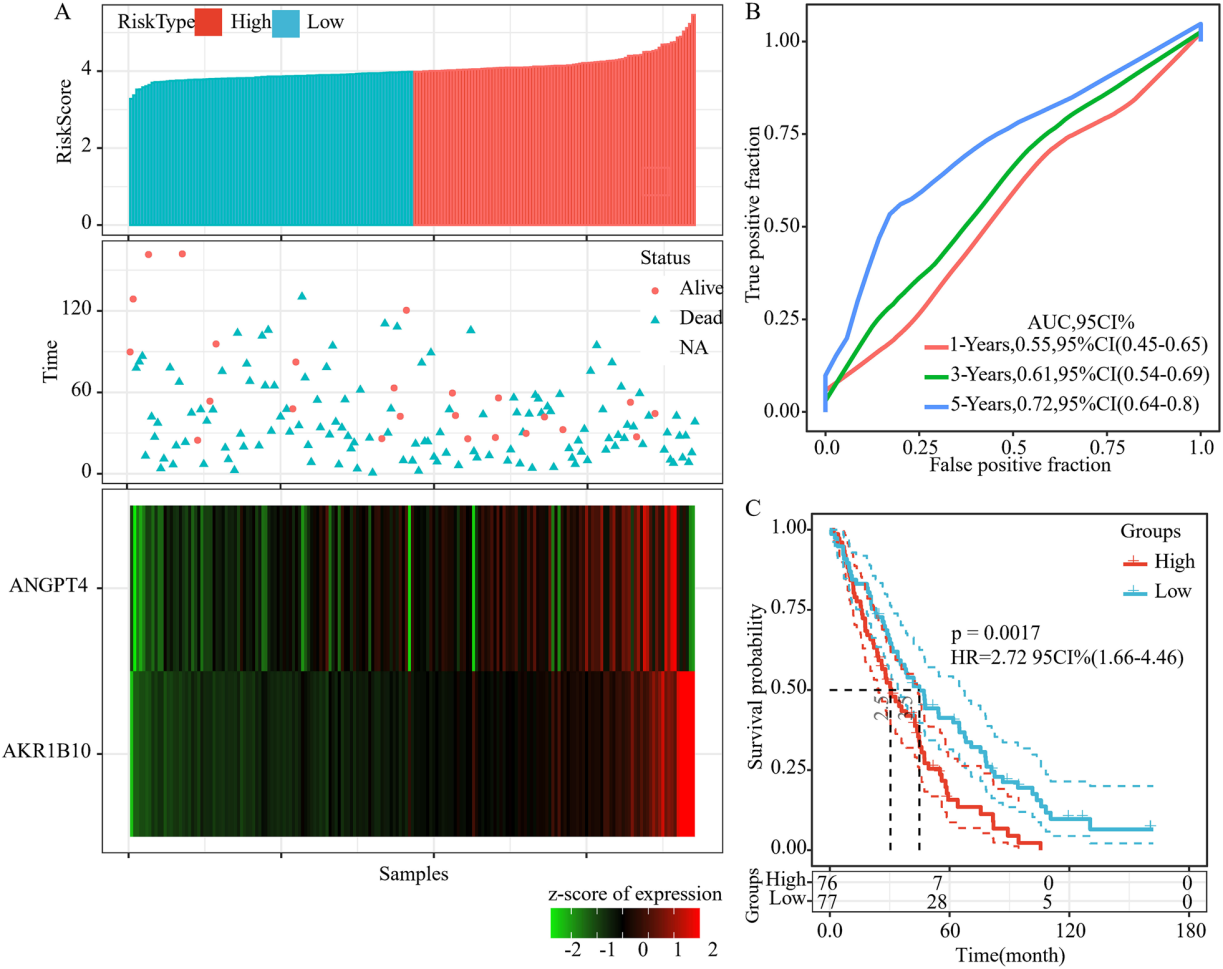
The relationship between risk score and prognosis in the GSE26712 set. **A** The relationship between 2-gene signature and survival status, survival duration, and expression in the GSE26712 set. **B** ROC and AUC of 2-gene signature in GSE26712 set. **C** KM survival curve of 2-gene signature in GSE26712 set

**Fig. 7:**
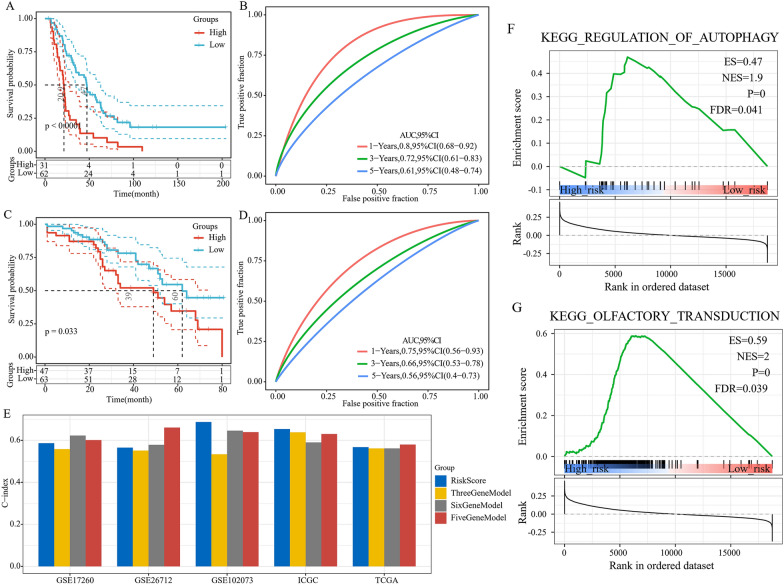
Analysis of pathway differences between high- and low-risk groups. **A** Enrichment results of REGULATION_OF_AUTOPHAGY. **B** Enrichment results of OLFACTORY_TRANSDUCTION

For the purpose of validating the performance of the 2-gene signature model, we computed the risk score of every sample in the TCGA test set and discovered that a higher risk score was associated with a shortened survival duration (Fig. 5A). Furthermore, the 2-gene signature had a 3-year AUC value of 0.6 (Fig. 5B). A remarkable difference in patient prognosis between the low- and high-risk groups was observed when the samples were categorized based on the median risk score (Fig. 5C). According to the results, the performance of the model showed consistency between the training set and TCGA test set. Among various data platforms, the GEO platform data set GSE26712 was used and served as the external data set so as to evaluate the classification performance of the 2-gene signature model. After computing the risk score for each sample in GSE26712, we discovered that a greater risk score was correlated with a shortened survival duration (Fig. 6A). Furthermore, the signature had a 3-year AUC value of 0.6 (Fig. 6B). A remarkable difference in patient prognosis between the low- and high-risk groups when the samples were subjected to classification based on the median risk score (Fig. 6C). The model's performance in the GSE26712 set was in line with the training set, according to these findings. Moreover, a set of ovarian cancer data set was acquired from the ICGC database. When the 2-gene signature model was used with ICGC-OV data set, the findings revealed that there was a remarkable prognostic difference between the high- and low-risk groups (Fig. 7A), and the signature’s 3-year AUC was 0.72 (Fig. 7B). Furthermore, to further verify the prognostic prediction of the 2-gene signature, GSE17260 was acquired from the GEO database. The findings showed that the prognosis of patients in the high-risk group was poorer compared to that in the low-risk group (Fig. 7C), and that the ROC analysis revealed a 3-year AUC of 0.66 (Fig. 7D). These findings indicated that the 2-gene signature was robust, with high prognostic performance across different validation queues.

### Clinical independence of the 2-gene signature model

The independence of the 2-gene signature model in a real-world scenario was investigated by performing multivariate and univariate Cox regression analysis to evaluate relevant HR, 95 percent confidence interval of HR, and *P*-value. We systematically analyzed the clinical data of patients in the training set, including STIC, age, stage, race, surgical outcome, and platinum status (Table 3). The multivariate and multivariate results revealed that the 2-gene signature showed significant correlation in both multivariate and univariate Cox regression analysis. Our model 2-gene signature was confirmed to be a prognostic indicator that is independent of other clinical factors, which could be applied in clinical practice.

**Table 3:** Univariate and multivariate Cox regression analyses identified clinical factors and clinical independence associated with prognosis

Variables	Univariate analysis			Multivariable analysis		
	HR	95%CI of HR	*P* value	HR	95%CI of HR	*P* value
Age	0.98	0.93–1.03	0.4356	0.9845	0.93–1.04	0.59
STAGE	1.53	0.67–3.5	0.3126	2.3353	0.96–5.66	0.06
RiskScore	2.72	1.63–4.52	0.00012	3.2963	1.82–5.95	7.7e−05

### Comparison with existing models

The predictive performance of the constructed 2-gene signature was subjected to a comparison with three previously produced gene signatures, namely, the 5-gene model of Zhang et al. [29], the 3-gene signature of Sergio Marchini et al. [30], and the 6-gene signature of Ma et al. [30]. When the same method was used to derive the risk scores of the samples by each model, the results demonstrated that according to their C-index distribution (Fig. 7E), the 2-gene signature showed a similar performance to the previous studies in the five datasets including the training set, the test set, and the external validation set, especially in GSE102073 and ICGC dataset. Thus, as compared with other signatures, the 2-gene signature with fewer genes involved showed a more convenient detection in clinical practice and had more application prospects.

### Analysis of pathway differences between high- and low-risk groups

In the training set, GSEA was used to analyze the pathways that were significantly enriched in both the high and low-risk groups, and two significantly enriched pathways REGULATION_OF_AUTOPHAGY and OLFACTORY_TRANSDUCTION were identified to be significantly activated in the high-risk samples (Fig. 7F, G).

## Discussion

Ovarian cancer is an extremely heterogeneous illness and patients with comparable TNM stages of ovarian cancer show different survival outcomes. Currently, demand for early screening to detected and treat ovarian cancer makes it difficult to predict individual outcomes using the conventional clinicopathological indicators, such as portal venous thromboembolism, vascular invasion, size of tumors, and TNM staging, especially in risk stratification, because “one-size-fits-all” treatment strategy has been found to be ineffective [31, 32]. The identification of prognostic molecular markers indicative of tumor biological characteristics has significance in the prevention and treatment of ovarian tumors. This study examined the expression profiles of the 822 HGSOC samples from five research cohorts of TCGA, ICGC, and GEO. We examined the OS of patients with KM curves in various data queues (Additional file 1: Fig. S1), although the median survival time was different. In general, apart from GSE102073 datasets, the overall survival rates of these datasets were similar. These differences may result from differences in living standards, medical conditions, such as varied follow-up periods. GSE102073 showed the optimal prognosis, while GSE102073 and GSE17260 had the shortest follow-up time. Variations in study cohorts, follow-up time, and environmental differences are always difficult to overcome in multi-data integration analysis, and due to the heterogeneity of tumors, these differences also have a great impact on the generalization ability of the model. Moreover, overfitting problems will also occur when different data sets are combined to form a large data set. Therefore, in this study, GSE102073 was selected as the training set, and the other four data sets served as the external validation set to evaluate the robustness and universality of the 2-gene model.

The functions of prognostic genes were analyzed with the aid of the R package Clusterprofiler to carry out GO and KEGG functional enrichment analysis on these 148 genes. The findings from the KEGG enrichment analysis confirmed that the genes were enriched to biological pathways such as fatty acid degradation, cholesterol metabolism, tyrosine metabolism, and the AMPK signaling pathway (Additional file 2: Fig. S2A). Biological process category, genes were mainly enriched to negative regulation of endopeptidase activity, organic hydroxy compound catabolic process, cholesterol transport, regulation of cholesterol transport, and other GO Terms (Additional file 2: Fig. S2B). Moreover, further study was performed to analyze the difference in the KEGG pathway between Cluster1 and Cluster2. The expression patterns of all the genes obtained in different KEGG pathways were analyzed by GSEA. Cluster1 samples with poor prognosis were significantly activated in the METABOLISM, DRUG METABOLISM CYTOCHROME P450, and Cluster2 samples with favorable prognosis were significantly activated in the CIRCADIAN RHYTHM MAMMAL pathway (Additional file 2: Fig. S2C).

Currently, gene signatures, such as Oncotype DX expressing 21 genes [33–35], and an 18-gene expression signature of coloprint in colon cancer, have been applied in clinical practice [36–38]. Gene expression profiling has evolved as a viable tool of high-throughput molecular identification for the purpose of identifying new prognostic indicators in cancer. Ding Q et al. [39] developed a 9-gene signature for evaluating the prognosis of patients with ovarian cancer by LASSO to analyze tumor microenvironment-associated genes. Wang R et al. [29] screened differentially expressed genes to develop a 5-gene signature, which was verified as an independent prognostic factor. Sun H et al. [40] identified 28 DNA repair genes related to the prognosis of patients with ovarian cancer by performing cluster analysis, univariate analysis, and stepwise regression. Although a variety of prognostic markers have been studied, there is currently a lack of prognostic markers directly available for ovarian cancer in clinical practice. The inclusion of multiple genes will increase detection troubles of a signature, which also proves the applicability and detection convenience of the 2-gene signature in clinical practice.

Tumor heterogeneity is one of the important reasons leading to different clinical outcomes of tumor patients. Therefore, there are molecular differences between different tumor patients than cell lines. LASSO is a dimension reduction method to find a relative optimal solution from high dimension to low dimension. Its principle also involves cross-validation and re-sampling. Therefore, different results will be obtained even if the same data set is used with the same LASSO method (known as “optimal solution” in the optimization method). This study used 100, 200, 500, 1000, 2000, 5000, 10,000 repetitions to perform LASSO regression on 80% of the samples randomly chosen from the training set, and analyzed the frequency of the top 10 genes with the greatest frequency (Additional file 3: Fig. S3). The results demonstrated that AKR1B10 and ANGPT4 genes showed the highest frequency in the seven repetitions.

AKR1B10 and ANGPT4 in our 2-gene signatures were risk factors. AKR1B10 is member B10 of Aldo–Keto Reductase family 1. The glycolysis ability of tumor cells with high-expressed AKR1B10 was reduced. Glucose is a cellular source, and an increase in oxidative utilization of fatty acids will enhance the metastasis and colonization of tumor cells [41]. AKR1B10 has been identified as a tumor proliferation and metastasis marker in multiple tumors, for example, AKR1B10 expression is predictive of the treatment response of locally advanced stomach cancer [42], and its expression is associated with poor prognosis and lymph node metastasis. Qi Wang et al. [43] found that serum expression of AKR1B10 is a diagnostic biomarker, as its expression is significantly up-regulated in patients with lung cancer that has metastasized to the brain, thus, determining the level of AKR1B10 can predict lung cancer patients with brain metastasis. Many experimental studies also proved AKR1B10 role in the pathogenesis of liver cancer, development, and resistance to chemotherapeutic drugs [44–46]. Oral squamous cell carcinoma patients with a high level of AKR1B10 in the saliva are often related to poor prognosis and progression [47]. AKR1B10 expression is remarkably downregulated in colorectal cancer, and its low expression is highly correlated with the unfavorable prognosis of patients with colorectal cancer [48]. These findings confirmed that the abnormal expression of AKR1B10 is closely associated with the occurrence and development of tumors. At present, the relationship between AKR1B10 expression and prognosis in HGSOC is rarely reported. The current findings confirmed that high-expressed AKR1B10 was related to a poor prognosis of HGSOC, and we also found that the high expression of ANGPT4, a member of the angiogenin family, led to an unfavorable prognosis of patients with HGSOC, which is consistent with the research conclusion proposed by Qin Yu et al. [49]. Regarding the expression of AKR1B10 and ANGPT4 genes, a 2-gene signature was established and verified to have the ability to stratify the prognosis of patients in the training set, TCGA test set, and the GEO verification set. GSEA revealed that the 2-gene signature-enriched pathway was strongly correlated with the pathways and biological processes involved in the occurrence and progression of tumors. These findings suggested that this model has clinical utility and can serve as a possible target for clinical patient diagnosis.

Nevertheless, several limitations remained. Firstly, a lack of certain clinical follow-up information excluded the possibility to take factors, including the existence of other health conditions of the patients, into consideration when distinguishing prognostic biomarkers. Secondly, the results acquired from bioinformatics analysis were not fully reliable, necessitating further experimental confirmation. Therefore, experimental and genetic studies involving a larger sample size and experimental verification need to be conducted in the future.

In this study, bioinformatics techniques were employed in this study for the purpose of identifying possible candidate genes for cancer prognosis from large samples. In conclusion, we constructed a 2-gene prognostic stratification system, with a low AUC in the validation and the training sets. The 2-gene signature was independent of clinical manifestations. Gene classifiers can optimize survival risk prediction compared with clinical characteristics. As a result, the adoption of the 2-gene signature as a molecular diagnostic test with a view of determining prognostic risk in patients with HGSOC could be promoted.

### Supplementary Information

**Additional file 1: Figure S1.** The overall survival of patients with KM curves in various data queues.

**Additional file 2: Figure S2.** Functional enrichment analysis of genes. **A** Enriched KEGG biological pathways. **B** Enriched GO terms in the “biological process” category. Different colors indicate different significance, and different sizes indicate the number of genes. **C** GSEA enrichment results of the KEGG Pathway in Cluster1 and Cluster2.

**Additional file 3: Figure S3.** LASSO regression of the frequency of the top 10 genes with the greatest frequency. **A** 100 repetitions to perform LASSO regression analyzed the frequency of the top 10 genes with the greatest frequency. **B** 200 repetitions to perform LASSO regression analyzed the frequency of the top 10 genes with the greatest frequency. **C** 500 repetitions to perform LASSO regression analyzed the frequency of the top 10 genes with the greatest frequency. **D** 1000 repetitions to perform LASSO regression analyzed the frequency of the top 10 genes with the greatest frequency. **E** 2000 repetitions to perform LASSO regression analyzed the frequency of the top 10 genes with the greatest frequency. **F** 5000 repetitions to perform LASSO regression analyzed the frequency of the top 10 genes with the greatest frequency. **G** 10,000 repetitions to perform LASSO regression analyzed the frequency of the top 10 genes with the greatest frequency. **H** 80,000 repetitions to perform LASSO regression analyzed the frequency of the top 10 genes with the greatest frequency.

## Data Availability

The datasets generated and/or analyzed during the current study are available in the [GSE102073] repository, [https://www.ncbi.nlm.nih.gov/geo/query/acc.cgi?acc=GSE102073]; in [GSE26712] repository, [https://www.ncbi.nlm.nih.gov/geo/query/acc.cgi?acc=GSE26712]. in [GSE17260] repository, [https://www.ncbi.nlm.nih.gov/geo/query/acc.cgi?acc=GSE17260].
