# Supplementary figures and images for: Development and validation of an individualized gene expression-based signature to predict overall survival of patients with high-grade serous ovarian carcinoma
Source: Eur J Med Res. 2023 Oct 27;28:465. doi: 10.1186/s40001-023-01376-0 (PMC10604403; doi:10.1186/s40001-023-01376-0)

Survival probability

Groups

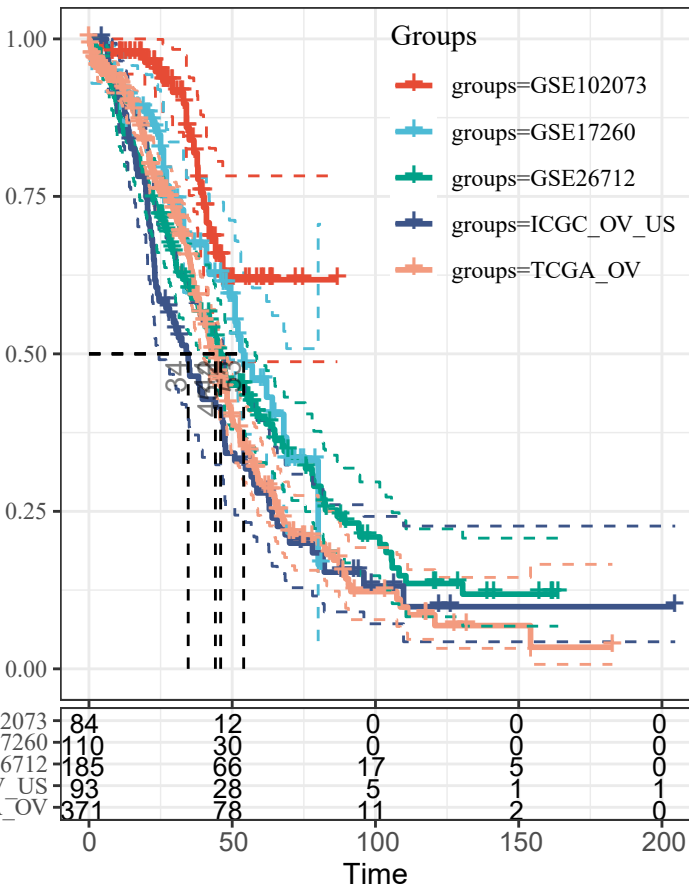

Supplement: Supplementary file 1 — Additional file 1: Figure S1. The overall survival of patients with KM curves in various data queues. [file 40001_2023_1376_MOESM1_ESM.pdf]

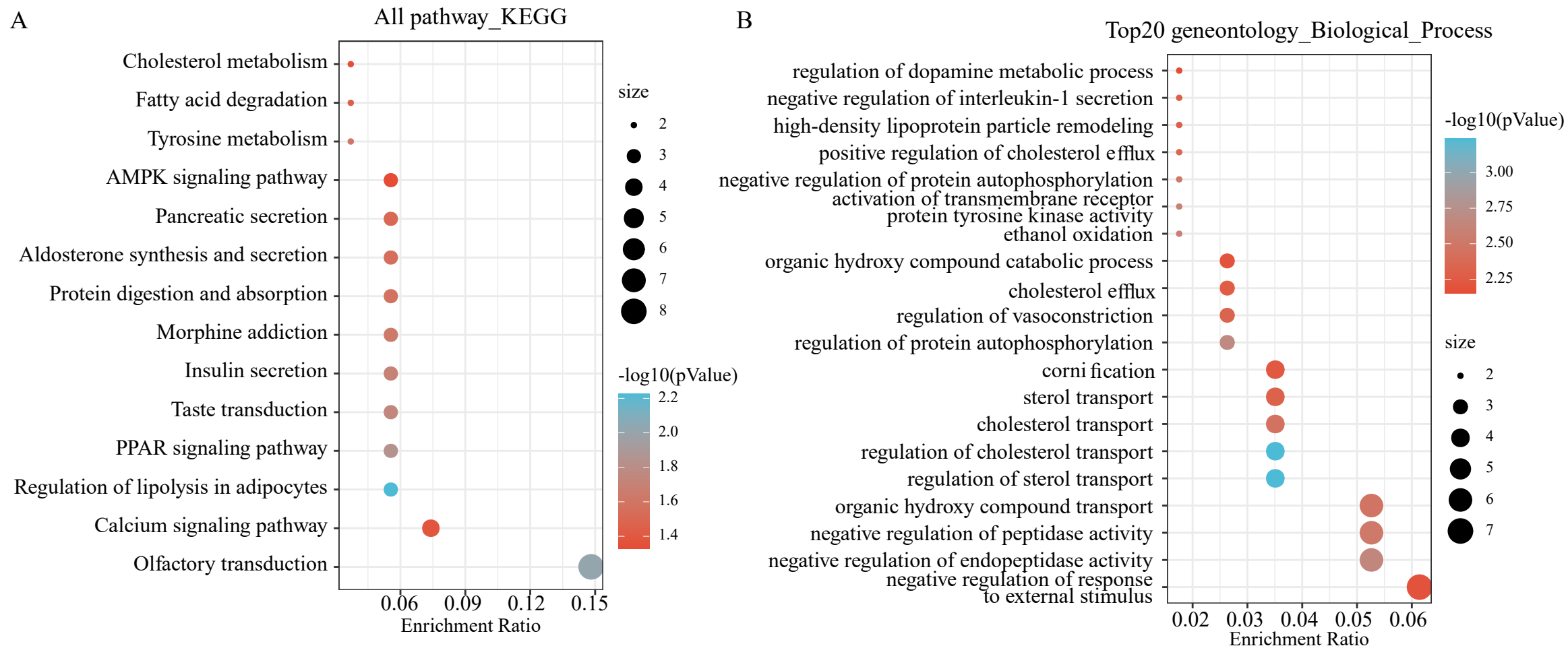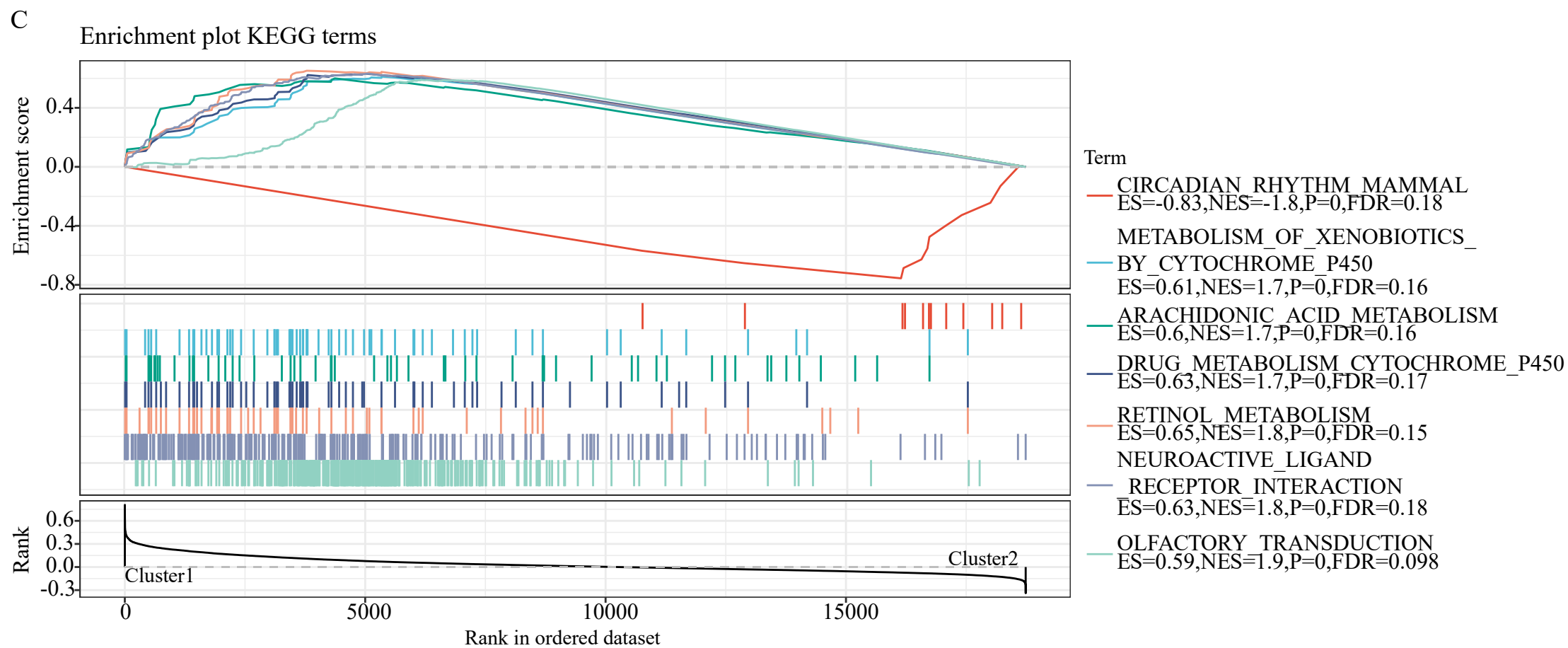

Supplement: Supplementary file 2 — Additional file 2: Figure S2. Functional enrichment analysis of genes. A Enriched KEGG biological pathways. B Enriched GO terms in the “biological process” category. Different colors indicate different significance, and different sizes indicate the number of genes. C GSEA enrichment results of the KEGG Pathway in Cluster1 and Cluster2. [file 40001_2023_1376_MOESM2_ESM.pdf]

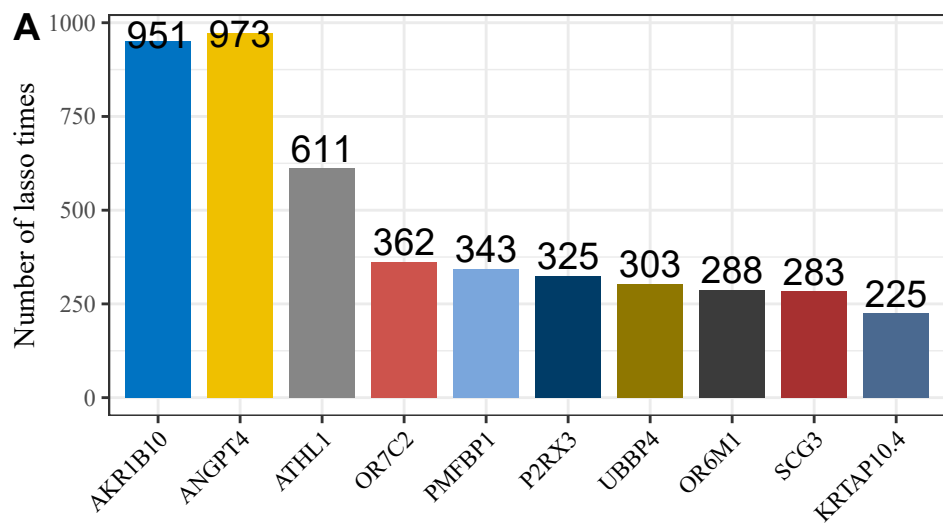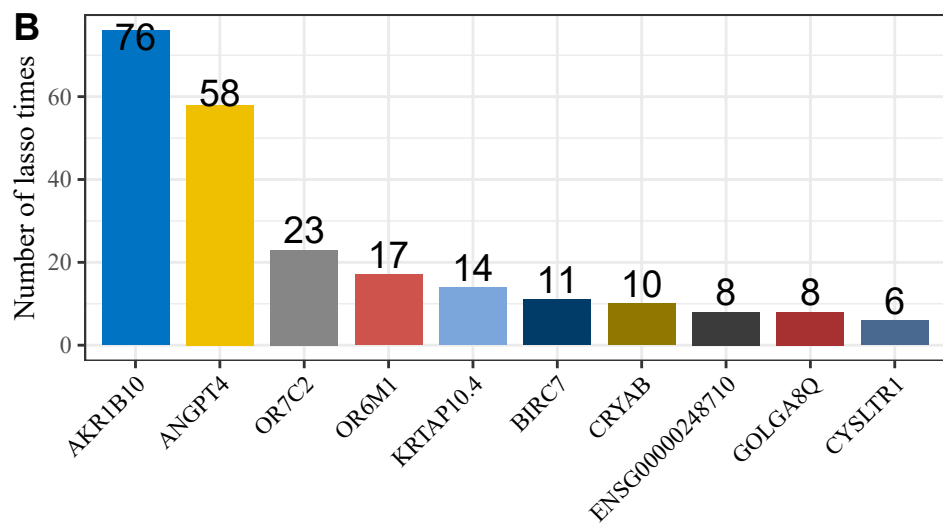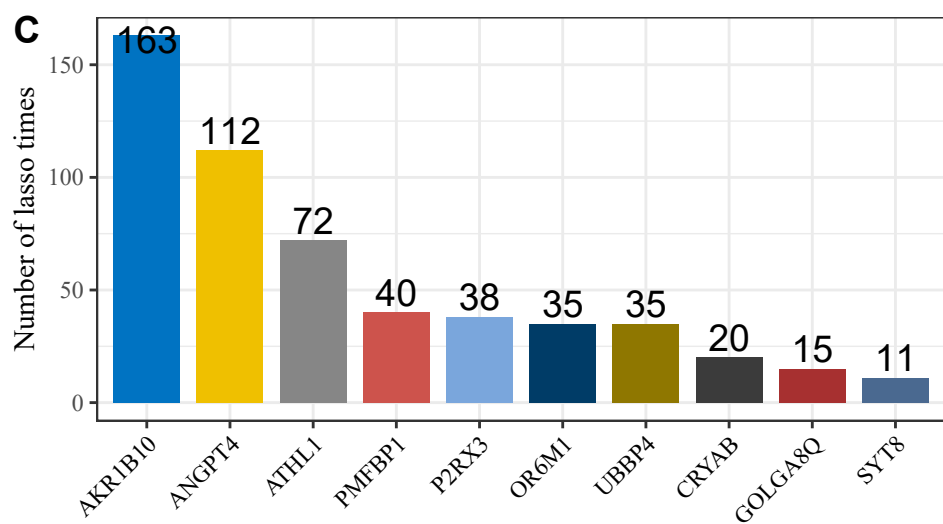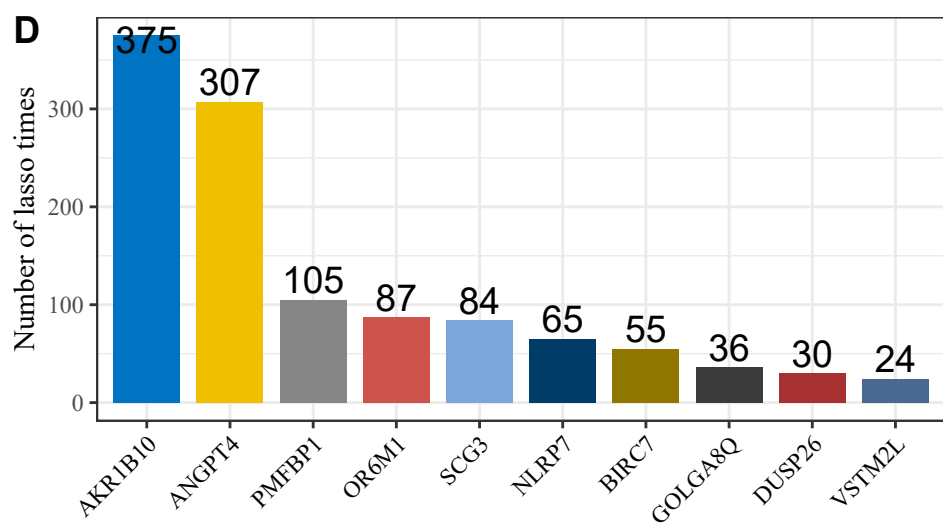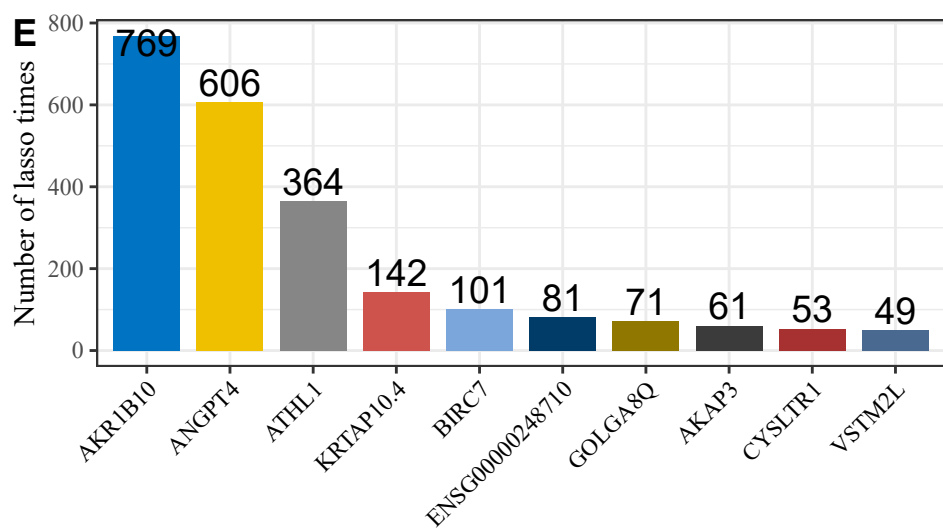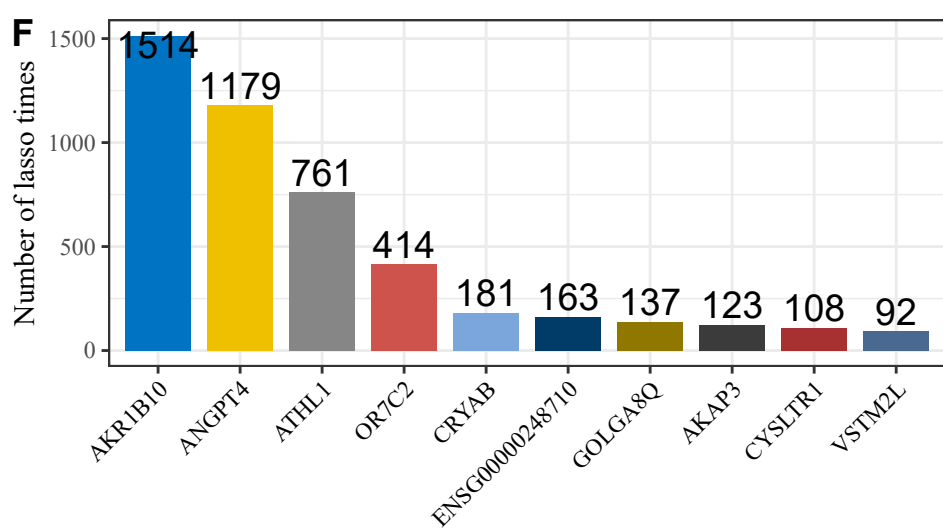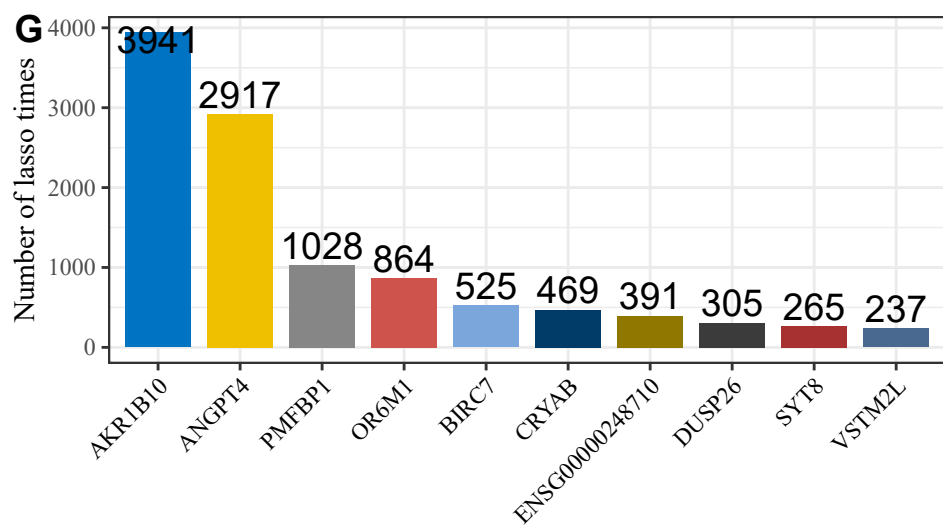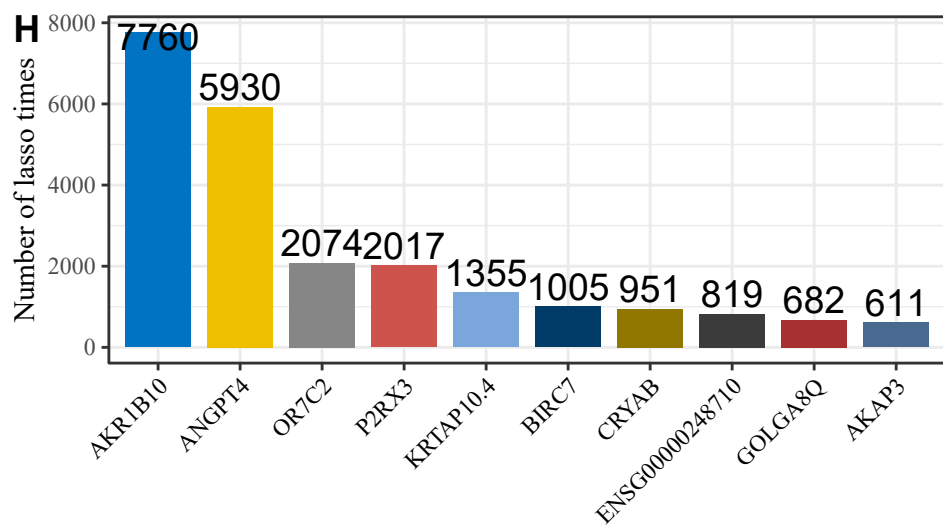

Supplement: Supplementary file 3 — Additional file 3: Figure S3. LASSO regression of the frequency of the top 10 genes with the greatest frequency. A 100 repetitions to perform LASSO regression analyzed the frequency of the top 10 genes with the greatest frequency. B 200 repetitions to perform LASSO regression analyzed the frequency of the top 10 genes with the greatest frequency. C 500 repetitions to perform LASSO regression analyzed the frequency of the top 10 genes with the greatest frequency. D 1000 repetitions to perform LASSO regression analyzed the frequency of the top 10 genes with the greatest frequency. E 2000 repetitions to perform LASSO regression analyzed the frequency of the top 10 genes with the greatest frequency. F 5000 repetitions to perform LASSO regression analyzed the frequency of the top 10 genes with the greatest frequency. G 10,000 repetitions to perform LASSO regression analyzed the frequency of the top 10 genes with the greatest frequency. H 80,000 repetitions to perform LASSO regression analyzed the frequency of the top 10 genes with the greatest frequency. [file 40001_2023_1376_MOESM3_ESM.pdf]
